# Underwater Acoustic Matched Field Imaging Based on Compressed Sensing

**DOI:** 10.3390/s151025577

**Published:** 2015-10-07

**Authors:** Huichen Yan, Jia Xu, Teng Long, Xudong Zhang

**Affiliations:** 1 Department of Electronic Engineering, Tsinghua University, Beijing 100084, China; E-Mails: yanhc11@mails.tsinghua.edu.cn (H.Y.); zhangxd@mail.tsinghua.edu.cn (X.Z.); 2 School of Information and Electronics, Beijing Institute of Technology, Beijing 100081, China; E-Mail: longteng@bit.edu.cn

**Keywords:** matched field processing (MFP), compressed sensing (CS), wave propagation, coherence parameter, coherence-excluding coherence optimized orthogonal matching pursuit (CCOOMP)

## Abstract

Matched field processing (MFP) is an effective method for underwater target imaging and localizing, but its performance is not guaranteed due to the nonuniqueness and instability problems caused by the underdetermined essence of MFP. By exploiting the sparsity of the targets in an imaging area, this paper proposes a compressive sensing MFP (CS-MFP) model from wave propagation theory by using randomly deployed sensors. In addition, the model’s recovery performance is investigated by exploring the lower bounds of the coherence parameter of the CS dictionary. Furthermore, this paper analyzes the robustness of CS-MFP with respect to the displacement of the sensors. Subsequently, a coherence-excluding coherence optimized orthogonal matching pursuit (CCOOMP) algorithm is proposed to overcome the high coherent dictionary problem in special cases. Finally, some numerical experiments are provided to demonstrate the effectiveness of the proposed CS-MFP method.

## 1. Introduction

Matched field processing (MFP) [1,2,3] is an attractive technique for imaging and localizing underwater targets, and its effectiveness has been widely verified in many real scenarios. Without emitting signals, it is known that the underwater targets can be recovered via MFP in the range-depth plane by using a sensor array passively. MFP collects sound pressure field data from targets and background and compares the data in the Bartlett processor with a series of pre-calculated data of all possible locations from models with greedy algorithms to finally determine the existence and positions of targets [4,5]. However, due to the limited number of sensors, the small array size and the complicated background, the recovery performance of existing MFP is not guaranteed in real application. Normally, the false MFP recoveries may occur on both number and positions for the targets.

These problems above are primarily owing to the fact that the MFP solves an underdetermined problem in practice [6]. To deal with this problem, the time-reversal technique [7] has been proposed as an alternative, but it also needs to be improved. As most MFP recovers a very small number of targets, this reminds us of the compressed sensing (CS) technique. To solve the underdetermined problem, the “small number”, or “sparsity”, property of the underwater targets in the scene should be effectively exploited. During the past decade, CS theories and techniques have been progressing at an overwhelming pace. For an underdetermined linear problem **y** = **Ax**, where **y** is a given observation and **A** is the so-called dictionary with far more columns than rows and each of its columns called atoms, it is known that the CS theory can provide a unique and stable solution as long as **x** is sparse and **A** satisfies the restricted isometrics property (RIP) condition. The RIP condition can be characterized by its coherence parameter [8,9,10,11], and the coherence parameter of **A** can be viewed as the largest normalized cross-correlation coefficient of the atom pairs. As computational imaging problems are mostly underdetermined, CS methods have been widely discussed and applied [6,12,13,14,15] in cases where imaging scene is sparse. CS imaging has been also proposed for MFP in [16], where it focused more on the computational complexity reduction rather than the imaging model and recovery performance.

We give a performance analysis of MFP from the view of CS. Instead of accelerating computation speed in Bartlett processing, in this paper, CS is hinged in the physical propagation model as [6] to improve the MFP performance. We focus on the shallow sea case and exploit the normal-mode acoustic propagation model to analyze the seabed and surface effects. In this case, the water is viewed as isotropic medium and the seabed and surface act as sound propagation boundaries. Boundaries affect the wave propagation, resulting in the problems of multi-path effects, interference effects, “blind spot effect” and so on [17]. According to wave propagation theory, the partial differential equation (PDE) is used for describing the propagation of sound in water from the wave equation, and the boundaries determine the constraints of the PDE directly. Based on the normal-mode acoustic propagation model, the shallow water is then regarded as a waveguide and a monochromatic wave is described as the sum of waves with different wave modes. Therefore, the MFP can be related to CS theory in essence and the CS-MFP model is proposed. In addition, this model can be easily transformed from the monochromatic one into a multi-frequency case.

In accordance with the RIP condition of CS theory, it is required that the atoms should be approximately orthogonal, or the coherence parameter of different atoms should be as small as possible. However, the dictionary in CS-MFP model cannot be designed arbitrarily. Furthermore, it is found that the dictionaries in CS-MFP are highly coherent by verifying the lower bound coherence parameter. This problem is related to the well-known off-grid problem and spectral CS (SCS) problem in CS recovery. In these problems, the nonzero elements of targets may not be exactly on the predefined grids, which will inevitably cause recovery errors. Nevertheless, refining the grids will generate the high coherent dictionaries. To solve this above problems, [18] and [19] discussed the off-grid CS recovery, and [20,21] proposed Spectral Iterative Hard Thresholding (SIHT) and Band-excluded Locally Optimized Orthogonal Matching Pursuit (BLOOMP) algorithms, respectively. BLOOMP can use fewer samples yet performed far better than SIHT. On the other hand, more complicated methods have been proposed recently for Fourier dictionaries [22,23]. In this paper, as the dictionary in CS-MFP is more complicated, we modify the BLOOMP method and provide a coherence-excluding coherence optimized orthogonal matching pursuit (CCOOMP) algorithm to solve the high coherent dictionary problem.

The contribution of this paper is as follows,

(1) The model of CS-MFP is established from wave propagation angle considering boundaries;
(2) The recovery performance of the model is discussed from a CS angle by examining the lower bound of the CS coherent parameter of the above model;
(3) The effective solution is given when targets are sparsely distributed.

The remainder of this paper is arranged as follows. In Section 2, the CS-MFP model is established for monochromatic wave cases. In Section 3, we propose a CCOOMP algorithm to solve the CS-MFP problem with high coherent dictionaries. In Section 4, we present some numerical experiments to verify the model and algorithm and extend the model to multi-frequency case. In Section 5, some conclusions are drawn.

## 2. Compressed Sensing MFP Model Formulation

This section describes the formulation of underwater acoustic CS-MFP imaging model in a shallow sea case from the angle of wave propagation. To articulate the model clearly, we consider simple boundaries in the formulation.

### 2.1. Wave Propagation Theories

**Figure 1:**
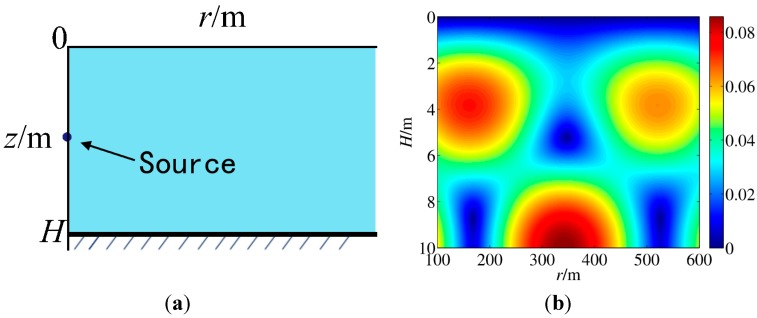
(**a**) The sketch of underwater circumstances; (**b**) The pressure field intensity generated by a source.

In propagation theory, a monochromatic acoustic wave generated from a point source in a boundless field will propagate in a form of spherical wave in the range-depth plane (*r*–*z* plane) according to the wave equation. As Figure 1a indicates, in a shallow sea case, assuming that sound propagation velocity c is a constant, sea surface (*z* = 0) is a soft boundary and seabed (*z* = H) is a hard boundary, the propagation of the acoustic wave can be modeled as a waveguide model in *r*–*z* plane. The propagation follows a wave equation and the two constraints for the wave equation are

(1) $$Z\left(n\right){|}_{z=0}=0,\;{\frac{\text{d}Z\left(n\right)}{\text{d}z}|}_{z=H}=0,\;\;\;1\leq n\leq N,$$

where Z(n) is the eigenfunction and N is the number of normal modes of the wave propagating in the water. Constrained by Equation (1), an acoustic source at $r\;=\;r_{0},\;z\;=\;z_{0}$, denoted as ($r_{0}$, $z_{0}$), with unit strength and an angle frequency ω will generate acoustic pressure field at a far-field position ( *r*, *z* ) as

(2) $$p\left(r,z\right)\;=-\frac{2\text{j}}{H}\sum_{n=1}^{N}\{\sqrt{\frac{2\pi }{\varsigma \left(n\right)(r-r_{0})}}sin\left(k_{z}\left(n\right)z\right)sin\left(k_{z}\left(n\right)z_{0}\right)\cdot exp\left[-\text{j}\left(\varsigma \left(n\right)(r-r_{0})-\frac{\pi }{4}\right)\right]\}$$

in which, $\text{j}=\sqrt{-1}$,

(3) $$N=\left\lfloor \frac{H\omega }{\pi c}+\frac{1}{2}\right\rfloor$$

where ⌊⋅⌋ is the ceiling operator and $k_{z}$, ς are vectors of length *N* representing the eigenvalues of wave modes,

(4) $$k_{z}\left(n\right)=\left(n-\frac{1}{2}\right)\frac{\pi }{H}$$

and

(5) $$\varsigma \left(n\right)=\sqrt{{\left(\frac{\omega }{c}\right)}^{2}-k_{z}{\left(n\right)}^{2}}$$

It can be shown that ς and $k_{z}$ are the horizontal and vertical components of the wave number ω/*c*. Figure 1b presents an example of the generated sound pressure field intensity from a source at position (0, *z*_0_) shown in Figure 1a as the point in the left. The reader can be referred to [24] for a full presentation of propagation theory.

### 2.2. CS-MFP Model Formulation

**Figure 2:**
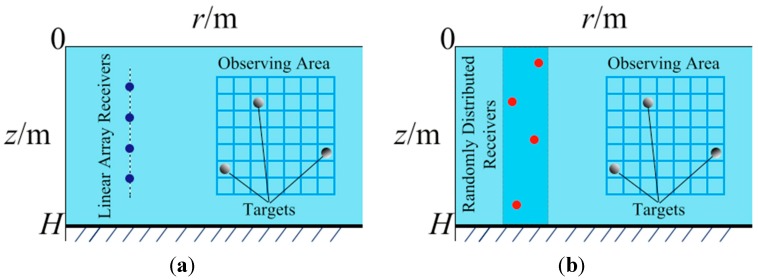
(**a**) Conventional MFP model Conventional MFP model; (**b**) CS-MFP model.

As Figure 2a shows, in the conventional MFP, a two-dimensional observation area of interest with hundreds of “candidate” pixels is determined in advance. For simplicity, we assume the targets only exist on a square lattice of dimension *Q* × *Q* with grid spacing Δr and Δz and denote the grid points’ coordinates as $r_{\text{T}}$, $z_{\text{T}}$). Hence, $r_{\text{T}}\left(i\right)=r_{\text{T}}\left(1\right)+\left(i-1\right)\Delta r,\;$$z_{\text{T}}\left(i\right)=z_{\text{T}}\left(1\right)+\left(i-1\right)\Delta z$, i =1, 2,···,Q. Matrix G denotes the strengths of the grid points to be determined, *i.e*., G(i, j) (i, j = 1, 2, ···, Q) represents the strength at coordinate $\left(r_{\text{T}}\left(i\right),z_{\text{T}}\left(j\right)\right)$. A uniformly linear sensor array with P elements is set in the far field at positions $\left(r_{\text{S}},z_{\text{S}}\right)$ to collect data denoted as y. The sensors can receive all the existing sound pressure generated by these targets. If we set $\left(r,z\right)=(r_{\text{S}},z_{\text{S}})$ and $\left(r_{0},z_{0})=(r_{\text{T}},z_{\text{T}}\right)$ in Equation (2), then p(r,z) can be also represented as $p(r_{\text{S}},z_{\text{S}}|r_{\text{T}},z_{\text{T}})$. Hence, y is a linear superposition of the entire sound pressure intensities generated from all the existing candidate pixels according to Equation (2) as

(6) y=A⋅vec(G)+e

where A represents the discrete version of Equation (2) and e is an observation error. With slight abuse of notations we have

(7) $$A\left(i,j\right)=p\left(r_{\text{S}}\left(i\right),z_{\text{S}}\left(i\right)|r_{\text{T}}\left(\left\lceil j/Q\right\rceil \right),z_{\text{T}}\left(j-\left(\left\lceil j/Q\right\rceil -1\right)Q\right)\right)$$

where $i=1,2,\;\cdot \cdot \cdot ,P,\;j=1,2,\cdot \cdot \cdot ,Q^{\text{2}}$, vec(·) is a function piling a matrix to a vector column by column. Denoting ${\left(\cdot \right)}^{\text{T}}$ as matrix transpose, we have

(8) $$\text{vec}\left(G\right)=\;{\left[G{\left(:,1\right)}^{\text{T}}\;G{\left(:,2\right)}^{\text{T}}\;\cdots \;G{\left(:,Q\right)}^{\text{T}}\right]}^{\text{T}}$$

Clearly, Equation (6) can be viewed as the weighted sum of the atoms in A with the weights representing the strengths of the targets.

In the conventional MFP framework, Bartlett processing is adopted therefrom to determine the most possible target positions among the candidate pixel positions. As the pixel number is greater compared to the number of sensors, it is obvious that the problem is underdetermined. Consequently, we obtain a linear model with the form of CS as

(9) y=Ax+e

In CS theory, **A** can be Gaussian, Bernoulli and partial Fourier random matrices, as they satisfy the RIP condition [10]. The randomness in these matrices plays a crucial role. In this new CS-MFP model, we propose to use a random distributed sensor array in a 2D plane instead of the traditional uniformly distributed linear array as Figure 2b plots. We will see the advantage of this setup of matrix **A** from the angle of compressed sensing in the following analysis, especially for resolution in r direction. In practice, the random array is also applicable. In fact, we can randomly deploy the sensors and keep track of their positions separately just as a linear array. In addition, we don’t have to stick to the uniform distance requirement. However, as Equation (7) indicates, it is not trivial to analyze the RIP or even for the coherence parameter of A. To comprehend the characteristics of A in Equation (7) to certain extent, let us separately observe each of the N components in Equation (2), *i.e*.,

(10) $$A=\sum_{n=1}^{N}A_{n}$$

and analyze the lower bound of the coherence parameter. Note in reality the separation is also possible by employing sound pressure sensor subarrays to form spatial bandpass filters for certain bandwidth at particular observation locations to form space filters. Denote the coherence parameter of A as μ. Here we use i, j instead of n to distinguish two subscriptions. According to the definition, the Gram matrix of **A** is

(11) $$\text{Gram}\left(A\right)=A^{\text{H}}A=\sum_{i=1}^{N}A_{i}^{\text{H}}\sum_{j=1}^{N}A_{j}=\sum_{i=1}^{N}A_{i}^{\text{H}}A_{i}+\sum_{i,j=1,i\neq j}^{N}A_{i}^{\text{H}}A_{j}$$

The coherence parameter of **A** is the maximum of the off-diagonal entry of Gram (**A**). Let us firstly analyze the first part of the right hand side of Equation (11). For a fixed i, two targets with the same $z_{0}$ but a Δr distance in r direction will generate two atoms with a correlation coefficient as

(12) $$\begin{array}{l} \mu_{r}\left(i,i\right)\\ =\frac{\left|{\left[\frac{1}{\sqrt{r}}sin\left(k_{z}\left(i\right)z\right)sin\left(k_{z}\left(i\right)z_{0}\right)exp\left(-\text{j}\varsigma \left(i\right)r\right)\right]}^{\text{H}}\left[\frac{1}{\sqrt{r+1\Delta r}}sin\left(k_{z}\left(i\right)z\right)sin\left(k_{z}\left(i\right)z_{0}\right)exp\left(-\text{j}\varsigma \left(i\right)\left(r+1\Delta r\right)\right)\right]\right|}{\left|\frac{1}{\sqrt{r}}sin\left(k_{z}\left(i\right)z\right)sin\left(k_{z}\left(i\right)z_{0}\right)exp\left(-\text{j}\varsigma \left(i\right)r\right)\right|\left|\frac{1}{\sqrt{r+1\Delta r}}sin\left(k_{z}\left(i\right)z\right)sin\left(k_{z}\left(i\right)z_{0}\right)exp\left(-\text{j}\varsigma \left(i\right)r+1\Delta r\right)\right|}\\ =\frac{\sum_{p=1}^{P}\frac{1}{\sqrt{r_{p}\left(r_{p}+\Delta r\right)}}{sin}^{2}\left(k_{z}\left(i\right)z_{p}\right)}{\sqrt{\sum_{p=1}^{P}\frac{1}{r_{p}}{sin}^{2}\left(k_{z}\left(i\right)z_{p}\right)}\sqrt{\sum_{p=1}^{P}\frac{1}{r_{p}+\Delta r}{sin}^{2}\left(k_{z}\left(i\right)z_{p}\right)}} \end{array}$$

On the other hand, two targets with the same r but a Δz distance in z direction will generate two atoms with a correlation coefficient

(13) $$\begin{array}{l} \mu_{z}\left(i,i\right)\\ =\frac{\left|{\left[\frac{1}{\sqrt{r}}sin\left(k_{z}\left(i\right)z\right)sin\left(k_{z}\left(i\right)\left(z_{0}+\Delta z\right)\right)exp\left(-\text{j}\varsigma \left(i\right)r\right)\right]}^{\text{H}}\left[\frac{1}{\sqrt{r}}sin\left(k_{z}\left(i\right)z\right)sin\left(k_{z}\left(i\right)z_{0}\right)exp\left(-\text{j}\varsigma \left(i\right)r\right)\right]\right|}{\left|\left[\frac{1}{\sqrt{r}}sin\left(k_{z}\left(i\right)z\right)sin\left(k_{z}\left(i\right)\left(z_{0}+\Delta z\right)\right)exp\left(-\text{j}\varsigma \left(i\right)r\right)\right]\right|\left|\left[\frac{1}{\sqrt{r}}sin\left(k_{z}\left(i\right)z\right)sin\left(k_{z}\left(i\right)z_{0}\right)exp\left(-\text{j}\varsigma \left(i\right)r\right)\right]\right|}\\ =\frac{\left|\sum_{p=1}^{P}\frac{1}{r_{p}}{sin}^{2}\left(k_{z}\left(i\right)z_{p}\right)sin\left(k_{z}\left(i\right)z_{0}\right)sin\left(k_{z}\left(i\right)\left(z_{0}+\Delta z\right)\right)\right|}{\sqrt{\sum_{p=1}^{P}\frac{1}{r_{p}}{sin}^{2}\left(k_{z}\left(i\right)z_{p}\right){sin}^{2}\left(k_{z}\left(i\right)\left(z_{0}+\Delta z\right)\right)}\sqrt{\sum_{p=1}^{P}\frac{1}{r_{p}}{sin}^{2}\left(k_{z}\left(i\right)z_{p}\right){sin}^{2}\left(k_{z}\left(i\right)z_{0}\right)}}=1. \end{array}$$

Now let us focus on the second part of Equation (11). Similar to the above discussion, we have for two matrix $A_{i}$ and $A_{j}$ (i≠j), two targets with the same r and a Δz distance in the z direction,

(14) $$\begin{array}{l} \mu_{z}\left(i,j\right)\\ =\frac{\left|\sum_{p=1}^{P}\frac{1}{\sqrt{\varsigma \left(i\right)\varsigma \left(j\right)}r_{p}}sin\left(k_{z}\left(i\right)z_{p}\right)sin\left(k_{z}\left(j\right)z_{p}\right)sin\left(k_{z}\left(i\right)z_{0}\right)sin\left(k_{z}\left(j\right)\left(z_{0}+\Delta z\right)\right)exp\left(-\text{j}\left[\varsigma \left(i\right)-\varsigma \left(j\right)\right]r_{p}\right)\right|}{\sqrt{\sum_{p=1}^{P}\frac{1}{\varsigma \left(i\right)r_{p}}{sin}^{2}\left(k_{z}\left(j\right)z_{p}\right){sin}^{2}\left(k_{z}\left(j\right)z_{0}\right)}\sqrt{\sum_{p=1}^{P}\frac{1}{\varsigma \left(j\right)r_{p}}{sin}^{2}\left(k_{z}\left(i\right)z_{p}\right){sin}^{2}\left(k_{z}\left(i\right)\left(z_{0}+\Delta z\right)\right)}}\\ =\frac{\left|\sum_{p=1}^{P}\frac{1}{r_{p}}sin\left(k_{z}\left(i\right)z_{p}\right)sin\left(k_{z}\left(j\right)z_{p}\right)exp\left(-\text{j}\left[\varsigma \left(i\right)-\varsigma \left(j\right)\right]r_{p}\right)\right|}{\sqrt{\sum_{p=1}^{P}\frac{1}{r_{p}}{sin}^{2}\left(k_{z}\left(i\right)z_{p}\right)}\sqrt{\sum_{p=1}^{P}\frac{1}{r_{p}}{sin}^{2}\left(k_{z}\left(j\right)z_{p}\right)}} \end{array}$$

For two targets with the same z and a Δr distance in the r direction,

(15) $$\begin{array}{l} \mu_{r}(i,j)\\ =\frac{\left|\sum_{p=1}^{P}\frac{1}{\sqrt{\varsigma \left(i\right)\varsigma \left(j\right)r_{p}\left(r_{p}+\Delta r\right)}}sin\left(k_{z}\left(i\right)z_{p}\right)sin\left(k_{z}\left(j\right)z_{p}\right)sin\left(k_{z}\left(i\right)z_{0}\right)sin\left(k_{z}\left(j\right)z_{0}\right)exp\left(-\text{j}\left[\varsigma \left(i\right)-\varsigma \left(j\right)\right]r_{p}\right)\right|}{\sqrt{\sum_{p=1}^{P}\frac{1}{\varsigma \left(i\right)r_{p}}{sin}^{2}\left(k_{z}\left(i\right)z_{p}\right){sin}^{2}\left(k_{z}\left(i\right)z_{0}\right)}\sqrt{\sum_{p=1}^{P}\frac{1}{\varsigma \left(j\right)\left(r_{p}+\Delta r\right)}{sin}^{2}\left(k_{z}\left(j\right)z_{p}\right){sin}^{2}\left(k_{z}\left(i\right)z_{0}\right)}}\\ =\frac{\left|\sum_{p=1}^{P}\frac{1}{\sqrt{r_{p}\left(r_{p}+\Delta r\right)}}sin\left(k_{z}\left(i\right)z_{p}\right)sin\left(k_{z}\left(j\right)z_{p}\right)exp\left(-\text{j}\left[\varsigma \left(i\right)-\varsigma \left(j\right)\right]r_{p}\right)\right|}{\sqrt{\sum_{p=1}^{P}\frac{1}{r_{p}}{sin}^{2}\left(k_{z}\left(i\right)z_{p}\right)}\sqrt{\sum_{p=1}^{P}\frac{1}{\left(r_{p}+\Delta r\right)}{sin}^{2}\left(k_{z}\left(j\right)z_{p}\right)}} \end{array}$$

Let us consider the case where *z_p_* is uniform distribution on [0, H] and *r_p_* is uniform distribution on [*r*_min_, *r*_max_]. Then we have for large *P*,

(16) $$\begin{array}{l} \;\sum_{p=1}^{P}\frac{1}{\sqrt{r_{p}\left(r_{p}+\Delta r\right)}}{sin}^{2}\left(k_{z}\left(i\right)z_{p}\right)=\sum_{p=1}^{P}\frac{1}{2\sqrt{r_{p}\left(r_{p}+\Delta r\right)}}\left[1-cos\left(2k_{z}\left(i\right)z_{p}\right)\right]\\ \;=\int_{r=r_{min}}^{r_{max}}\int_{z=0}^{H}\frac{1}{2\sqrt{r\left(r+\Delta r\right)}}\left[1-cos\left(\pi \frac{2i-1}{H}z\right)\right]\text{d}z\text{d}r\approx \frac{H}{2}\ln\left(\frac{r_{max}+\frac{1}{2}\Delta r}{r_{min}+\frac{1}{2}\Delta r}\right) \end{array}$$

(17) $$\begin{array}{l} \;\sqrt{\sum_{p=1}^{P}\frac{1}{r_{p}}{sin}^{2}\left(k_{z}\left(i\right)z_{p}\right)}=\sqrt{\sum_{p=1}^{P}\frac{1}{2r_{p}}\left[1-cos\left(2k_{z}\left(i\right)z_{p}\right)\right]}\\ \;=\sqrt{\int_{r=r_{min}}^{r_{max}}\int_{z=0}^{H}\frac{1}{2r}\left[1-cos\left(\pi \frac{2i-1}{H}z\right)\right]\text{d}z\text{d}r}=\sqrt{\frac{H}{2}\ln\left(\frac{r_{max}}{r_{min}}\right)} \end{array}$$

(18) $$\begin{array}{l} \sqrt{\sum_{p=1}^{P}\frac{1}{r_{p}+\Delta r}{sin}^{2}\left(k_{z}\left(i\right)z_{p}\right)}\;=\sqrt{\sum_{p=1}^{P}\frac{1}{2\left(r_{p}+\Delta r\right)}\left[1-cos\left(2k_{z}\left(i\right)z_{p}\right)\right]}\\ \;=\sqrt{\int_{r=r_{min}}^{r_{max}}\int_{z=0}^{H}\frac{1}{2\left(r+\Delta r\right)}\left[1-cos\left(\pi \frac{2i-1}{H}z\right)\right]\text{d}z\text{d}r}=\sqrt{\frac{H}{2}\ln\left(\frac{r_{max}+\Delta r}{r_{min}+\Delta r}\right)} \end{array}$$

According to Equations (16)–(18),

(19) $$\mu_{r}\left(i,i\right)=\frac{\ln\left(r_{max}+\frac{1}{2}\Delta r\right)-\ln\left(r_{min}+\frac{1}{2}\Delta r\right)}{\sqrt{\ln\left(\frac{r_{max}}{r_{min}}\right)\ln\left(\frac{r_{max}+\Delta r}{r_{min}+\Delta r}\right)}}$$

in Equation (12). Similarly, $\mu_{z}\text{(}i,j\text{)=0}$ and $\mu_{r}\text{(}i,j\text{)=0}$ in this case.

From the above discussion, we have four conclusions.

(1) the distance Δz has no impact on the coherence parameter.
(2) the random deployment of sensors helps lower the coherence parameter in Equations (12) and (15), according to Cauchy-Schwarz inequality. Traditionally, if we use uniform array, we will have ${\tilde{\mu }}_{r}\left(i,i\right)=1$ in Equation (12) and greater ${\tilde{\mu }}_{r}\text{(}i,j\text{)}$ as
  $${\tilde{\mu }}_{r}(i,j)=\sum_{p=1}^{P}sin\left(k_{z}\left(i\right)z_{p}\right)sin\left(k_{z}\left(j\right)z_{p}\right)/\sqrt{\sum_{p=1}^{P}{sin}^{2}\left(k_{z}\left(i\right)z_{p}\right)}\sqrt{\sum_{p=1}^{P}{sin}^{2}\left(k_{z}\left(j\right)z_{p}\right)}$$
(3) in the special case, the high coherent part is mainly contributed by signals of the same modes.
(4) if all the $A_{n}$ (n=1, 2,⋯,N) are compensated to have equal energy, we have a lower bound
  (20) $$\begin{array}{l} \mu \geq max\left(\mu_{r},\mu_{z}\right)\\ \;=\frac{1}{N}max\left(\mu_{r}\left(i,i\right),\mu_{z}\left(i,i\right)\right)=\frac{1}{N} \end{array}$$

According to [25], if the target number is less than

(21) $$\left(\mu^{-1}+1\right)/2,$$

then x in Equation (9) can be recovered uniquely using greedy algorithms. This Theorem determines the performance of CS-MFP model.

### 2.3. Robustness of Coherence

In this subsection, we discuss the robustness of the coherence parameter. We analyze the correlation coefficient change w.r.t. the displacement of the sensors. As the sensors are deposed in the flowing water, this discussion becomes necessary. When there is a Δz displacement in the z direction, for the nth component in Equation (2), the correlation coefficient between the new measurement and the theoretical measurement can be expressed as

(22) $$\mu_{z}=\frac{\left|{\left[\text{sin}\left(k_{\text{z}}\left(n\right)z\right)\right]}^{\text{T}}\left[\text{sin}\left(k_{\text{z}}\left(n\right)\left(z\text{ + }1\Delta \text{z}\right)\right)\right]\right|}{{\left\|\text{sin}\left(k_{\text{z}}\left(n\right)z\right)\right\|}_{2}{\left\|\text{sin}\left(k_{\text{z}}\left(n\right)\left(z\text{ + }1\Delta \text{z}\right)\right)\right\|}_{2}}$$

When vector z is set randomly, the sensors are uniformly distributed in the z direction, we have

(23) $$\mu_{z}^{2}=\frac{{\left[\sum_{p=1}^{P}\text{cos}\left[\text{2}k_{\text{z}}\left(n\right)z_{p}\text{ + }k_{\text{z}}\left(n\right)\Delta \text{z}\right]-P\text{cos}\left(k_{\text{z}}\left(n\right)\Delta \text{z}\right)\right]}^{2}}{\left[P-\sum_{p=1}^{P}\text{cos}\left(\text{2}k_{\text{z}}\left(n\right)z_{p}\right)\right]\left[P-\sum_{p=1}^{P}\text{cos}\left(\text{2}k_{\text{z}}\left(n\right)\left(z_{p}\text{ + }\Delta \text{z}\right)\right)\right]}$$

The summation is approximated as integral for large P. Hence, the numerator can be simplified as

(24) $$\begin{array}{l} P^{2}{\text{cos}}^{2}\left(k_{z}\left(n\right)\Delta z\right)-\frac{P\text{cos}\left(k_{z}\left(n\right)\Delta z\right)}{k_{z}\left(n\right)}\left\{sin\left[\text{2}k_{z}\left(n\right)H\text{ + }k_{z}\left(n\right)\Delta z\right]-sin\left[k_{z}\left(n\right)\Delta z\right]\right\}\\ +\frac{2cos\left[\text{2}k_{z}\left(n\right)H\text{ + 2}k_{z}\left(n\right)\Delta z\right]-cos\left[4k_{z}\left(n\right)H\text{ + 2}k_{z}\left(n\right)\Delta z\right]-cos\left[\text{2}k_{z}\left(n\right)\Delta z\right]-2cos\left[\text{2}k_{z}\left(n\right)H\right]+2}{8k_{z}{\left(n\right)}^{2}} \end{array}$$

And the denominator is

(25) $$\begin{array}{l} P^{2}-\frac{P}{\text{2}k_{z}\left(n\right)}sin\left(\text{2}k_{z}\left(n\right)H\right)-\frac{P}{\text{2}k_{z}\left(n\right)}sin\left(\text{2}k_{z}\left(n\right)H\right)\\ +\frac{1}{4k_{z}{\left(n\right)}^{2}}sin\left(\text{2}k_{z}\left(n\right)H\right)\left[sin\left(\text{2}k_{z}\left(n\right)\left(H+\Delta z\right)\right)-sin\left(\text{2}k_{z}\left(n\right)\Delta z\right)\right] \end{array}$$

Hence, we have

(26) $$\mu_{z}=\left|cos\left(k_{z}\left(n\right)\Delta z\right)\right|$$

This result indicates the robustness deteriorates with the increase of n. On the other hand, the robustness w.r.t. r has the same form of Equation (12).

### 2.4. Multi-Frequency CS-MFP Case

In model Equation (6), the angle frequency of the targets is fixed as ω. It is a reasonable assumption as in real applications the bandwidths of the transducers are relatively narrow. However, in some wide band or multi-frequency cases, the model can be extended. Besides a two-dimensional search in r-z space, frequency dimension is added as the third dimension, whose dimensionality is denoted as R. In this way, we obtain a more intricate matrix A to replace A in Equation (9) with

$$A=\;\left[A_{\omega_{1}}\;A_{\omega_{2}}\;\cdots \;A_{\omega_{R}}\right]$$

Similarly, we can add even more dimensions to the model, for instance, a time series search. However these dimensions deteriorate the RIP of the dictionary, making it more difficult to accurately recover the targets with the same number of sensors.

## 3. CS-MFP Recovery with High Coherent Dictionaries via CCOOMP

**Figure 3:**
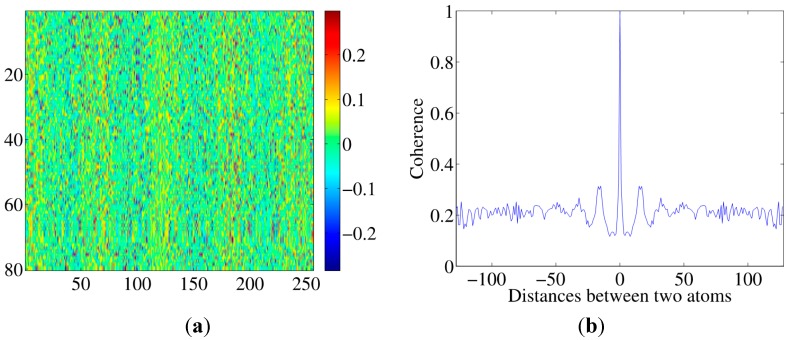
(**a**) A real part of the matrix **A** example; (**b**) **A**’s average correlation coefficients.

However, as it can be seen from Equations (12)–(15) and the simulations in Section 4, in general, it is very difficult to give an explicit expression of μ. It also may be high. This is a common problem faced in CS imaging applications, as in conventional CS theories matrix A can be meticulously designed, whereas in imaging applications, A is linked to physical quantities without much design freedom as Equation (6) shows. This problem resembles an off-grid problem in CS and spectral CS problems. Off-grid problems occur universally in CS imaging applications [13,14,15,21,26], as in reality, targets are not distributed exactly as the lattice is divided, or a discretized dictionary cannot fully express an analog signal $\hat{x}$ from Equation (9). BLOOMP is a successful tool to solve this problem. In these problems, high coherent atoms form a neighborhood or the so-called “band”, whereas in this problem, the high coherent atoms are randomly distributed in the dictionary (see Figure 1b and Figure 3b). We then modify the BLOOMP method to solve this problem.

Assuming 0<η<1, we first define the η-coherence index set of atom k to be the set

(27) $$C_{\eta }\left(k\right)=\left\{i|\mu \left(i,k\right)>\eta \right\}$$

and the η-coherence index set of an index set S is hence defined as

(28) $$C_{\eta }\left(S\right)=\cup_{k\in S}C_{\eta }\left(k\right)$$

and

(29) $$C_{\eta }^{2}\left(S\right)=C\left(C_{\eta }\left(S\right)\right)$$

Here we assume the targets are not distributed on positions with correlation coefficient larger than η, or for any i∈supp{x},

(30) $$i\cap C_{\eta }^{2}\left(\text{supp}\left(x\right)\backslash i\right)=\emptyset$$

Then, we obtain the Coherence-excluding Coherence Optimized Orthogonal Matching Pursuit (CCOOMP) method as Algorithm 1 shows. CCOOMP is similar to BLOOMP [21] except in the implementing, CCOOMP virtually excludes the atoms according to the correlation coefficients rather than the physical distances. Coherence Optimization (CO) (Algorithm 2) is embedded in CCOOMP.

**Algorithm 1** Coherence-Excluding Coherence Optimized Orthogonal Matching Pursuit (CCOOMP)
1: **Input:** A, y, η>0, s, t, ep
2: **Initialization:** $x_{0}=0,\;r=y$ and $S^{0}=Ø$ 
3: **Iteration:** For n=1,⋯,s
4: $i_{max}=\arg\underset{i}{max}\left|\langle r^{n-1},A\left(:,i\right)\rangle \right|,\;i\notin C_{\eta }^{2}\left(S^{n-1}\right)$
5: $S^{n}=\text{CO}\left(S^{n-1}\cup \left\{i_{max}\right\}\right)$
6: $x^{n}=\arg\underset{z}{min}{\left\|Az-y\right\|}_{2}\;s.t.\text{ supp}\left(z\right)\in S^{\text{n}}$
7: $r^{n}=y-Ax^{n}$
8: **return:** $x^{s}$

**Algorithm 2** Coherence Optimization (CO)
1: **Input:** $A,\;y,\;\eta >0,\;S^{0}=\left\{i_{1},\;i_{2},\cdots ,\;i_{k}\right\}$
2: **Iteration:** For n=1,⋯,k
3: $x^{n}=\arg\underset{i}{max}{\left\|\text{Ax}-\text{b}\right\|}_{2},\text{ supp}\left(x\right)=\left(S^{n-1}\backslash \left\{i_{n}\right\}\cup \left\{j_{n}\right\}\right),\;j_{n}\in C_{\eta }\left(\left\{i_{n}\right\}\right)$
4: $S^{n}=\text{supp}\left(x^{n}\right)$
5: **return:** $S^{k}$

## 4. Numerical Experiments and Performance Analysis

In this section, numerical experiments are conducted to demonstrate the proposed model and algorithm. We still consider a shallow sea case. In this case *H* = 200 m. We are going to observe an area at a distance *r* ∈ [100 m, 132 m] and depth *z* ∈ [2 m, 17 m] with 16 × 16 pixels. Range resolution $\rho_{r}$ is set 2 m, depth resolution ρ*z* = 1 m. We uniformly randomly place 80 transducers along *r* ∈[0 m, 10 m] and *z* ∈[0, H], and the central frequency of the transducers is 6 kHz. Note according to Equation (3), *N* = 25. Then matrix A is determined. Figure 3a illuminates an example of real part of matrix A. In Figure 3b we calculate the normalized correlation coefficients of each atom with all the other atoms in the dictionary and average it. In this case, high correlation coefficients occur randomly rather than forming a neighbor band around 0 as in SCS. This is a result of the scattering of the boundaries.

**Figure 4:**
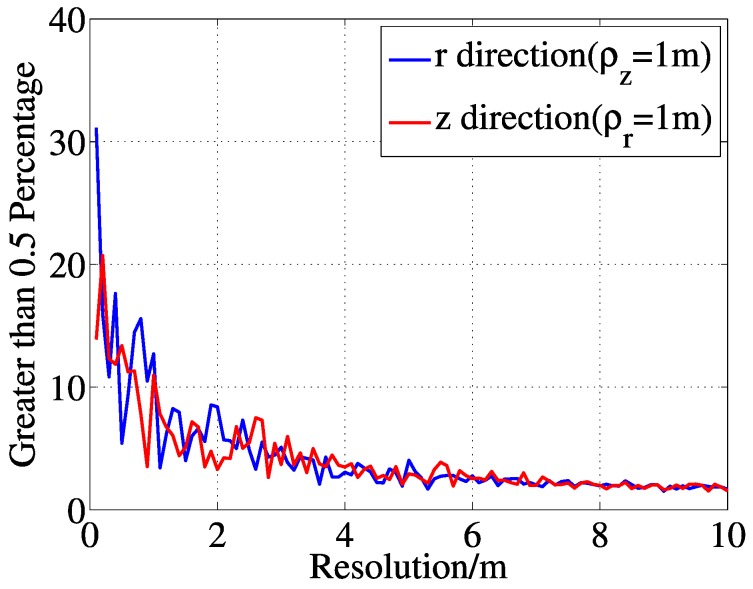
Percentage of high coherent atoms (coefficient ≥ 0.5) in the dictionary.

To obtain a better understanding of the dictionary, we analyze the coherence property of the matrix w.r.t., the resolution in *r*–*z* dimensions. In this particular experiment, the average percentages of high coherent atoms are drawn in Figure 4 after 10 iterations. As can be expected, the pairs of high coherent atoms increase with the resolution of imaging. Now, from the above experiment, we can see the high coherence of the dictionaries.

**Figure 5:**
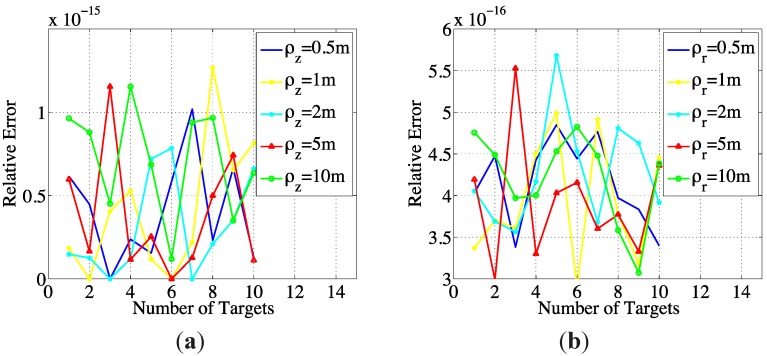
(**a**) Relative imaging errors w.r.t. numbers of targets under different depth resolutions with ρ*_r_* = 2 m fixed; (**b**) Relative imaging errors w.r.t. numbers of targets under different range resolutions with ρ*_z_* = 2 m fixed.

In the next experiment, the CCOOMP algorithm is applied to the imaging process. The sparse targets are set randomly in the observing area with coherence no greater than 0.5. For comparison, Figure 5 shows the imaging errors with CCOOMP in terms of number of targets under different resolutions. For each number of targets, 100 simulations are performed and the average error is plotted. It is seen that by properly setting the coherent parameter, the recovery result can be satisfactory.

**Figure 6:**
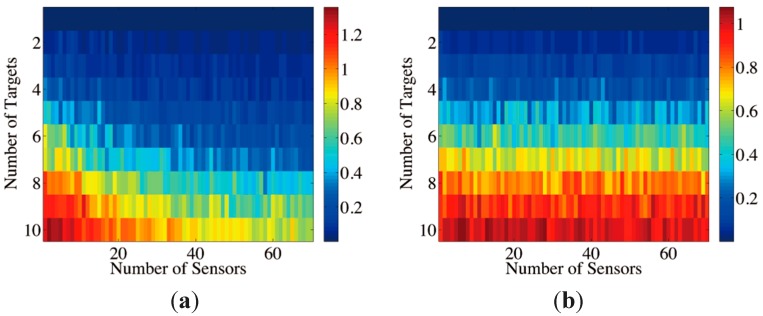
(**a**) Recovery errors w.r.t. numbers of targets and sensors with CS-MFP model; (**b**) Recovery errors w.r.t. numbers of targets and sensors with traditional MFP model.

In the next experiment, the numbers of sensors and targets change simultaneously, and the targets are set with random positions and phases regardless of the coherence. These targets are recovered with the CS-MFP sensor model and the traditional MFP model. The average relative square errors of 100 trials are shown in Figure 6. With greater number of targets, the CS-MFP model outperform traditional model overwhelmingly.

**Figure 7:**
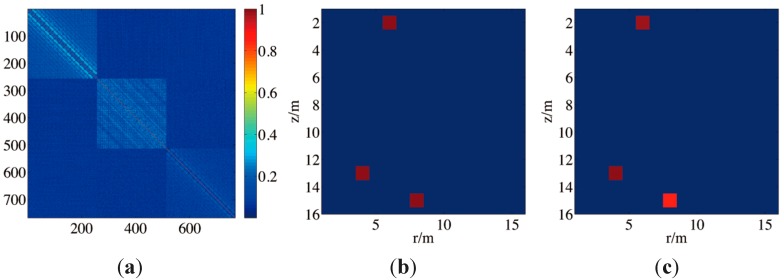
(**a**) A Gram matrix of A in a multi-frequency case; (**b**) The targets of interest; (**c**) The recovery result.

In the next experiment, the multi-frequency case is simulated. In the experiment, three frequencies 4 kHz, 8 kHz, 16 kHz are chosen as the frequency grids. The numbers of normal modes are 16, 32 and 67 respectively. Figure 7a presents a picture of the Gram matrix of matrix A. Three blocks are clearly seen along the diagonal. As can be seen, the high coherent atoms are distributed mainly in atoms with the same frequency. Hence, expanding dictionary will not obviously worsen the coherence parameter in this case. CS is applicable nevertheless. Figure 7b,c presents a result. Three targets with different frequencies are generated in the area of interest, and they are recovered with CCOOMP.

**Figure 8:**
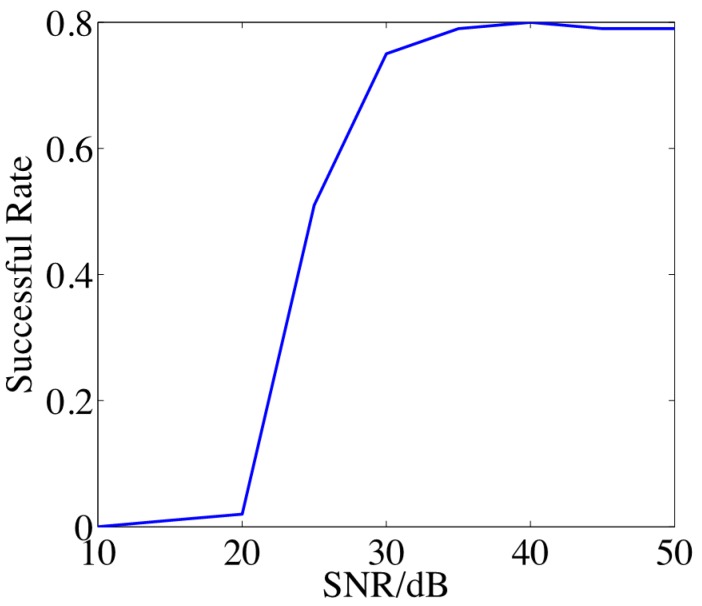
The successful recovery rate in terms of SNR.

In the last experiment, we examine the performance of the algorithm w.r.t. noise. In this experiment, two targets are placed randomly with random amplitudes and phases. The coherence of the targets is less than 0.5. The measurement noise is assumed as White Gaussian noise. We use bottleneck distance as the measurement, *i.e*., the recovery is counted as a success when the support of **x** is recovered. Figure 8 shows the successful recovery rate in terms of signal to noise ratio (SNR) in dB with 100 trials in each case. From Figure 8, with cases of SNR > 30 dB, good performance can be obtained. This constraint applies to the sea with low Beaufort scales and strong underwater targets.

## 5. Conclusions

Based on acoustic propagation in medium with boundaries, this paper presented a CS-MFP model in the application of underwater sparse target imaging and localizing. By exploiting the sparsity of targets in underwater environment, the feasibility of CS method in MFP application is discussed. Both the coherence of the constructed matrix and the robustness of the coherence w.r.t. the displacement of the sensors were analyzed. However, the direct application of CS may result in a high coherence parameter problem. The proposed CCOOMP algorithm was effective in solving high coherent dictionary recovery problems, especially when an atom's high coherent atoms do not form a neighborhood around it. Finally, numerical experiments were provided to demonstrate the effectiveness of the proposed CS-based MFP method.
